# Visual activities of daily living survey for retinitis pigmentosa

**DOI:** 10.1007/s10384-024-01122-x

**Published:** 2024-09-23

**Authors:** Takeshi Sugawara, Kyongsun Pak, Gen Miura, Takayuki Baba

**Affiliations:** 1 The Department of Ophthalmology, Chiba University Hospital, 1-8-1 Inohana, Chuo-ku, Chiba, 260-8677 Japan; 2 The Translational Research and Development Center (TRAD), Chiba University Hospital, 1- 8-1 Inohana, Chuo-ku, Chiba, 260-8677 Japan; 3 The Department of Data Science, National Center for Child Health and Development (NCCHD), Ookura 2-10-1, Setagaya-ku, Tokyo, 157-8535 Japan

**Keywords:** Retinitis pigmentosa, Visual ADL (activities of Daily Living), LVFAM (the low-vision-specific function and activities of daily living measure), Patient reported Outcome (PRO)

## Abstract

**Purpose:**

To gauge the value of low-vision-specific function and activities of daily living measures (LVFAM) in the assessment of Patient Reported Outcomes (PRO) of Retinitis Pigmentosa (RP).

**Study design:**

Prospective observation study.

**Methods:**

The May-July 2023 Visual activities of daily living (ADL) Survey of the low-vision-specific function and activities of daily living measure (LVFAM) was conducted on 15 RP patients attending our Ophthalmology outpatient clinic. We used the better corrected visual acuity, and the better Mean Deviation (MD) values of the central 10 degrees of Humphrey’s visual field as macular sensitivity, and examined the total scores after Rasch Analysis for each of the low-vision-specific function of Daily Living (LVFDL) and the low-vision-specific activities of Daily Living (LVADL) among LVFAM, as well as the relationship between the items.

**Results:**

Age 26–78 (mean 60.8), 5 men and 10 women, corrected visual acuity of 0.01-1.0 (mean 0.45) in the better eye, macular sensitivity of 3.47-40.00 (mean 18.60) dB, mean 52.2 for LVFDL and 66.4 for LVADL. The correlations were positive for visual acuity and LVFDL and LVADL, and negative for macular sensitivity, LVFDL and LVADL. In addition, four items were scored 0 by at least 5 (33%) of the LVFDL respondents, and two items were scored 1 by at least 5 (33%) of the LVADL respondents, suggesting that the results differed by question items.

**Conclusion:**

The LVFAM was useful not only for the total score but also for each question item in the development strategy of PROs in RP with no treatment.

## Introduction

Retinitis Pigmentosa (RP) is a chronic progressive disease for which there is no treatment, and in typical cases, visual acuity is impaired after the peripheral visual field is narrowed [1]. The area of the peripheral visual field in the Goldmann visual field test and electroretinogram (ERG), which are routinely performed in RP, are not considered standard measures in clinical studies. It is necessary to develop some sort of evaluation scale as well as treatment methods. Patient Reported Outcomes (PRO) are recommended in clinical trials in all therapeutic areas, and patient-oriented evaluation criteria have been recently emphasized in many clinical research fields [2–5]. Ophthalmological research reports the advantages of ADL in assessing treatment for AMD and cataracts [6]. This review emphasizes the importance of including an assessment of ADLs in patients undergoing IOL insertion, summarizes IOLs designed for insertion in patients with AMD, and describes how appropriate patient selection, proper visual assessment, including assessments related to quality of life, and pre- and postoperative care can improve outcomes. Recently, an article proposing the introduction of the low-vision-specific function and activities of daily living measures (LVFAM) has been published in Japan [7]. After creating the tentative version, Ono et al. surveyed 186 low vision patients on the tentative index, added a Rasch analysis, and developed the LVFAM. In this study, we applied the LVFAM to RP patients, investigated its relationship to corrected visual acuity and macular sensitivity, and discuss the development of PROs for RP.

## Subjects and methods

From May to July 2023, after approval by the Ethics Committee (#M10497) of Chiba University Hospital (UMIN 000050635), 15 RP patients aged 20 to 80 years (corrected visual acuity of 0.01 to 1.0 in each eye, excluding cases with ocular diseases other than refraction) attending the outpatient department of the Chiba University Hospital were asked, after obtaining written consent, to complete the survey items in order of simplicity. The low-vision-specific function of Daily Living (LVFDL) questionnaire, which consists of 23 items (question 1–23) on a 3-point scale (0: invisible, 1: sometimes invisible, 2: visible), was assessed by the LVFAM, and the low-vision-specific activities of Daily Living (LVADL), which consists of 9 items (question 1–9) on a 4-point scale (0: not active because it is difficult to see, 1: hard to be active because it is difficult to see, 2: hard to see, but active, 3: active without problems), was also assessed by the LVFAM, asking whether the patient had actually performed the activities. The LVFAM results were first correlated with the logMAR transformed value of the better corrected visual acuity; the LVFAM results were then correlated with the absolute values of the better Humphrey visual field (HFA) mean deviation (MD) value of the central 10 degrees as macular sensitivity. The relationship between each item and the total score after Rasch Analysis were examined. The Spearman correlation coefficient was used for statistical purposes. Note that the LVFAM itself is not included in this paper as a reference, since only a Japanese-language version is available. The Japanese version of the LVFAM can be found at,https://kaken.nii.ac.jp/ja/file/KAKENHI-PROJECT-24500607/24500607seika.pdf.

## Results

The subjects who underwent LVFAM in this study ranged in age from 26 to 78 years (mean 60.8 years), 5 men and 10 women, with corrected visual acuity of 0.01 to 1.0 (logMAR equivalent mean 0.61) in the better eye, and MD values of -3.47 to 40.00 (mean − 18.60) at 10 degrees center, with an average LVADL was 66.4 and mean LVFDL was 52.2 after Rasch Analysis.

**Fig. 1:**
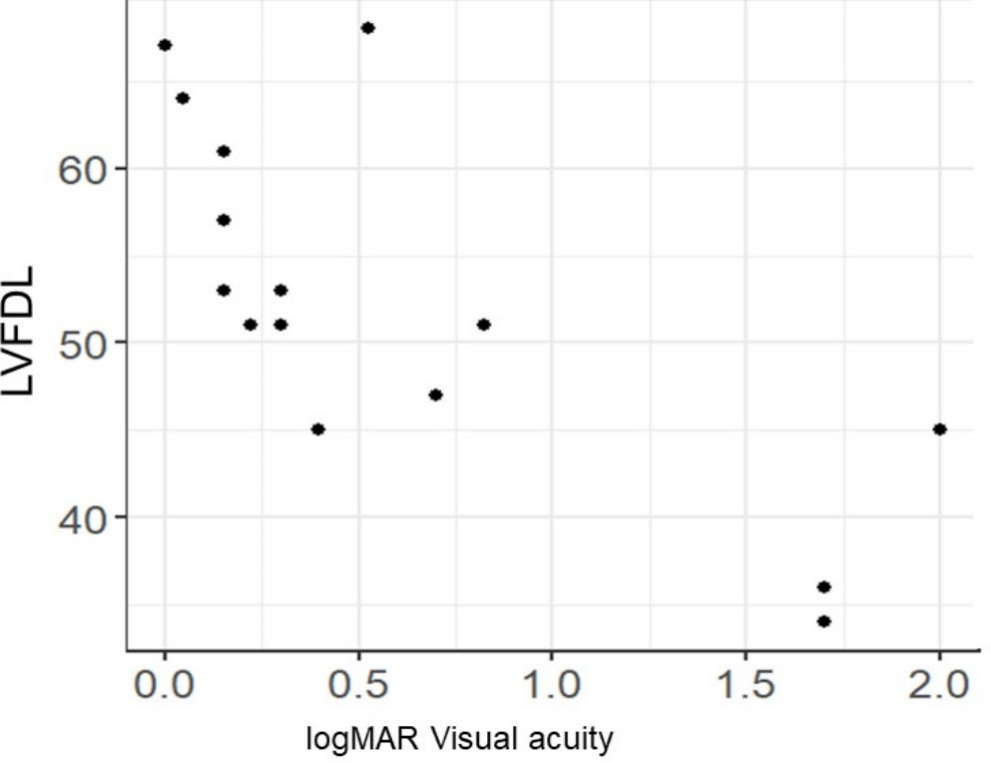
LVFDL and logMAR visual acuity. Spearman’s correlation is -0.76 (-0.92 to -0.41), Statistical difference is *p* < 0.0001. (LVFDL: the low-vision-specific function of Daily Living)

**Fig. 2:**
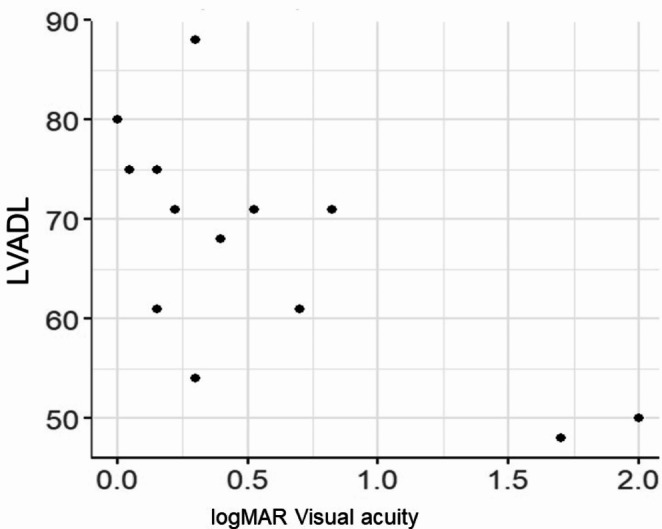
LVADL and logMAR visual acuity. Spearman’s correlation is -0.73 (-0.90 to -0.34), Statistical difference is *p* = 0.002. (LVADL: the low-vision-specific activities of Daily Living)

**Fig. 3:**
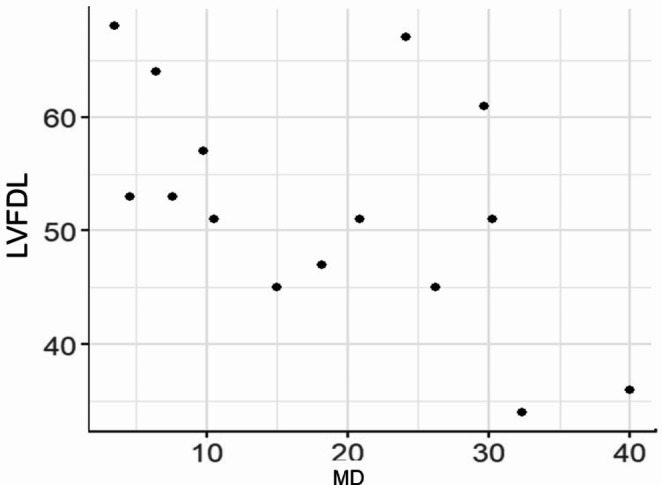
LVFDL and macular sensitivity (MD). Spearman’s correlation is -0.60 (-0.85 to -0.13), Statistical difference is *p* = 0.018

**Fig. 4:**
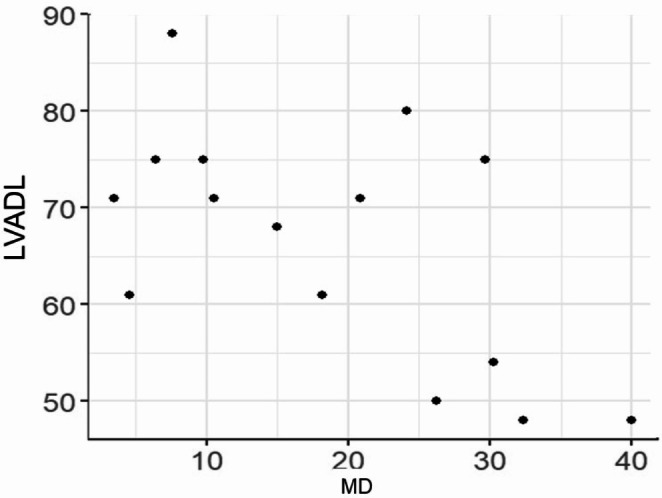
LVADL and macular sensitivity (MD). Spearman’s correlation is -0.52 (-0.81 to -0.01), Statistical difference is *p* = 0.048

The correlation coefficients of logMAR visual acuity and negative Spearman’s correlation coefficient for each of LVFDL and LVADL after Rasch Analysis and the better eye are shown in the scatterplot, suggesting a negative correlation with logMAR visual acuity for both LVFDL; -0.76 (-0.92 - -0.41), *p* < 0.0001 and LVADL; -0.73 (-0.90 - -0.34), *p* = 0.002, especially LVFDL, which was well correlated with corrected visual acuity (Figs. 1 and 2). Although the degree of visual field narrowing was not examined in this situation, corrected visual acuity affected visual ADL even in RP. The association between LVFAM and macular sensitivity (-MD value) after Rasch Analysis is shown in Spearman’s correlation coefficient and scatter plots, suggesting a negative correlation with -MD value for both LVFDL; -0.60 (-0.85 - -0.13), *p* = 0.018 and LVADL; -0.52 (-0.81 - -0.01), *p* = 0.048 (-MD value, macular sensitivity, indicates worse absolute value) (Figs. 3 and 4). Although the situation does not reflect the degree of peripheral visual field impairment, the results suggest that macular sensitivity affects visual ADL.

Examining each question item, among the 15 subjects, more than 5 (33%) responded with a score of 0 (difficult to obtain information (invisible)) on the LVFDL for 4 items: question 13 (letters on price tags, etc.), question 14 (price list/time table) and questions 21, 22, 23 (price list/time table, appearance of person across the room, appearance of person across the street), which were assumed to be particularly difficult to see in daily life for RP patients. Two items, question 6 (going out) and question 8 (writing), were assumed to be particularly difficult daily life activities for RP patients; these were answered by more than 5 (33%) of the LVADLs as 1 or less (being active or inactive due to visual difficulty) .

## Discussion

The criteria for evaluating efficacy and the timing of evaluation have been a constant point of contention with the regulatory Authority in Clinical Trials and have not been changed to date in either drugs or devices for RP patients [8, 9]. Many RP cases are chronic, progress slowly, and do not progress for about one year, so clinical trials over several years are needed, but for various reasons long term clinical trials are difficult to conduct. In addition, it is difficult to expect improvement because the disease often progresses irreversibly. Even in two-arm comparative studies with placebo or sham control groups, it can be assumed that neither the control nor the treatment group will worsen or improve visual function over the course of a one- to two-year study.

Visual function includes visual acuity and visual field as well as macular sensitivity. Macular sensitivity was defined as the -MD value when tested in the central 10-degree mode of the static quantitative HFA. However, macular sensitivity is not routinely tested even in clinical practice, and it is unclear what significance it has from the patient’s perspective, and its relationship with quality of life related to conventional visual functions has been investigated in RP patients [10, 11]. Visual ADL data is needed as well as visual QOL information.

What significance it has from the patient’s perspective, and its relationship with quality of life related to conventional visual function has not been investigated. The LVFAM used in this study as a visual ADL test is a questionnaire developed from the original LVFAM for patients with low vision. Generally, low vision is defined as corrected visual acuity of less than 0.3, but even if the corrected visual acuity is 1.0, patients with RP and progressive visual field narrowing are considered to be in need of a low vision clinic, and were included in the target population. The results of the LVFAM show that in the daily life of RP patients, most difficult to see were letters on price tags and tags of products, price lists and timetables, and the appearance of people a short distance away, while the most difficult activities were going out and writing. Since LVFAM was correlated with visual acuity, the visual ADL study LVFAM may reflect visual function in RP as well. Since macular sensitivity also correlated with LVFAM, the negative MD value of 10 degrees of HFA, defined as macular sensitivity, could reflect PRO as a visual function other than corrected visual acuity.

Some ALD studies in specific situations of patients with some form of visual dysfunction are reported [12, 13]. On the other hand, using spectacle-mounted prisms for field expansion in 15 patients with retinitis pigmentosa (RP) provided visual field expansion and spatial orientation benefits [14]. However, these are different from the LVFAM used in this study, so comparisons are not applicable. Ono, Suzukamo, and colleagues in Japan collected items from domestic and international literature on ADL measurements in low vision patients and questionnaires in similar fields, and compared them with the content of the Activities’ item of the International Classification of Functional Living (ICF) to create a tentative version of the index. The LVFAM was then completed by calculating the level of difficulty and fit statistics for each item and examining the degree of fit to the Rasch model in 186 low vision patients [7]. In this paper, it was performed on all patients independent of the primary disease and included 36 RPs. However, in the final results, items characteristic of RP related to visual field were excluded from the results as they need to be further examined. Therefore, we felt that more RP cases needed to be further examined.

The LFVAM took nearly 30 min if the patient was unfamiliar with the test, but once the they had practiced and became familiar with it, the test could usually be performed within 10 min, or 15 min at the most. Those who were more cautious about individual questionnaire items tended to take longer, and the examination time varied depending on age and whether the patient came to the hospital with a family member.

The limitation of this study is that the number of cases was limited to 15, but the total score of each LVFAM was related to corrected visual acuity and macular sensitivity, and there was a trend in the evaluation index for each question. This may be expected as a breakthrough in terms of fostering an evaluation system for the development of PRO in diseases for which a cure has not yet been established.

In the development of treatments for RP, the primary endpoint is both important and not easy to set [8, 9]. This is because RP is a disease of visual field narrowing in which central visual acuity is preserved to the end. Macular sensitivity was established as a visual field that approximates central visual acuity, but its clinical significance is unknown because it has not been standardized in clinical practice. Therefore, it is necessary to confirm the relationship of macular sensitivity with visual QOL and visual ADL, which are directly related to PRO. This is one of a number of reports of cases of visual field impairment such as RP with a focus on PROs [15, 16]. Visual QOL is important in addition to visual ADL, and should be examined with various PROs. Opinions may differ because assessing the results takes much time. Furthermore, as time passes, each PRO should be updated with more appropriate PROs. Based on the results of this study, further research on LVFAM for RP is warranted in the future. It is expected that an English version of the LVFAM will be prepared in the future to investigate trends in other populations as well.
